# Supplementary material for: Partially sintered copper‒ceria as excellent catalyst for the high-temperature reverse water gas shift reaction
Source: Nat Commun. 2022 Feb 14;13:867. doi: 10.1038/s41467-022-28476-5 (PMC8844362; doi:10.1038/s41467-022-28476-5)
Supplement: Supplementary file 1 — Supplementary Information [file 41467_2022_28476_MOESM1_ESM.pdf]

Supplementary Information for

Partially sintered copper–ceria as excellent catalyst for the high-temperature reverse water gas shift reaction

Hao-Xin Liu[1], Shan-Qing Li[2], Wei-Wei Wang[1], Wen-Zhu Yu[1], Wu-Jun Zhang[3], Chao Ma[3]*, Chun-Jiang Jia[1]*

[1]Key Laboratory for Colloid and Interface Chemistry, Key Laboratory of Special Aggregated Materials, School of Chemistry and Chemical Engineering, Shandong University, Jinan, 250100, China.
[2]Key Laboratory of Micro-Nano Powder and Advanced Energy Materials of Anhui Higher Education Institutes, Chizhou University, Chizhou, 247000, China
[3]College of Materials Science and Engineering, Hunan University, Changsha, 410082, China.
These authors contributed equally: Hao-Xin Liu, Shan-Qing Li.
*Corresponding author: jiacj@sdu.edu.cn; cma@hnu.edu.cn

Table of Contents

Supplementary Methods

Supplementary Figures

Supplementary Tables

Supplementary References

Supplementary Methods:

Preparation of CeO_2 supports: The CeO_2 nanorods were prepared by hydrothermal method[1]. 1.3 g of $Ce(NO_3)_3{\cdot}6H_2O$ was dissolved in 20 mL of Millipore water and the solution was added to an aqueous of NaOH solution under vigorous stirring. After that, the mixture was heated at 100 °C for 24 h. The obtained precipitates were separated by centrifugation, washed with deionized water and ethanol, and then dried at 60 °C overnight. The ceria nanoparticles were prepared as previous report[1]. With the assistance of CTAB, $Ce(NO_3)_3{\cdot}6H_2O$ was precipitated by NaOH until pH at 9 and aged at 90 °C for 3 h. The suspension was centrifuged, washed, and dried to be further use. The preparation of CeO_2 nanocube was similar to a previous study[2]. 0.868 g of $Ce(NO_3)_3{\cdot}6H_2O$ and 8.4 g NaOH were dissolved in 5 and 35 mL of deionized water, respectively. After that, the mixture was heated at 180 ºC for 24 h. The precipitates were separated by centrifuged, washed with deionized water and ethanol, followed by drying at 60 ºC for 12 h.

Preparation of copper–aluminum Catalyst: Except for the support, the experimental procedures were consistent with the preparation of copper–ceria catalyst.

Preparation of Al_2O_3 nanobelt: 3.22 g of $Al(NO_3)_2{\cdot}9H_2O$ and 4.6 g of $CO(NH_2)_2$ were added to 60 mL of deionized water. Then, the above solution was transferred to a Teflon bottle. Next, the Teflon bottle was then put in a autoclave and kept at 100 °C for 48 h. The obtained precipitates were separated by centrifugation, washed with deionized water for three times and ethanol for one time, and then dried at 80 °C for 10 h. Finally, the obtained solid was calcined in still air at 600 °C for 2 h (heating rate: 1 °C/min).

X-ray Diffraction (XRD): The *ex situ* X-ray Diffraction (XRD) were tested on PANalytical X'pert3 powder diffractometer (40 kV, 40 mA, $\lambda_{Cu\text{-}K\alpha}$ = 0.15406 nm). The diffraction patterns of *ex situ* XRD were collected from 10° to 90° with test time of 15 min.

Inductively Coupled Plasma-Atomic Emission Spectroscopy (ICP-AES): For all catalysts, the ICP−AES measurements were carried out on an IRIS Intrepid II XSP instrument (Thermo Electron Corporation).

Brunauer-Emmett-Teller (BET) Analysis: The surface area was measured on Builder SSA-4200 physic-sorption analyzer. Specific surface area was calculated by using the Brunauer-Emmett-Teller method.

H_2 temperature-programmed reduction (H_2-TPR): Hydrogen temperature Programmed reduction

(H_2-TPR) was carried out using Builder PCSA-1000 instrument equipped with a thermal conductivity detector (TCD). The fresh catalysts (30 mg, 20–40 mesh) were activated in high pure air at 300 °C for 30 min, then cooled to room temperature followed by heating the samples from room temperature to 600 °C with the ramping rate of 10 °C min^{-1} in the presence of 5% H_2/Ar flowing at the rate of 30 mL min^{-1}.

CO_2 temperature-programmed desorption (CO_2-TPD), CO_2 dissociation experiment, temperature-programmed surface reaction (TPSR): All these experiments were performed on a lab-made reactor. The outlet gases were recorded by mass spectrum (LC-D200M, TILON). The catalysts were firstly reduced in 5% H_2/Ar at 600 °C for 60 min, and then flushed with Ar gas flow (30 mL min^{-1}) at room temperature for 30 min. For CO_2-TPD, the catalysts were saturated with 2% CO_2/Ar (30 mL min^{-1}) at room temperature for 30 min followed by purging with Ar gas flow (30 ml min^{-1}) for 30 min to clear away all the physical adsorbed CO_2 molecules. And then the CO_2-TPD experiment was started from room temperature to 600 °C with a ramping rate of 10 °C min^{-1} under Ar gas flow (30 mL min^{-1}). For CO_2 dissociation experiment, after H_2 activation and Ar gas purge, the samples were flushed with 2% CO_2/Ar with heating from room temperature to 600 °C. For TPSR, the samples were treated with mixed gas with 23% CO_2 and 69% H_2 with heating from room temperature to 600 °C.

X-ray photoelectron spectroscopy (XPS): The XPS measurements were carried out at an Axis Ultra XPS spectrometer from Kratos, Japan. The operation was under 225W of accelerating voltage and Al Kα radiation. The C 1s signal located at 284.8eV was used to calibrate each spectra for accurate binding energies.

***In situ* diffuse reflectance infrared Fourier transform spectroscopy (DRIFTS):** All of the DRIFTS spectra were collected by using a Bruker Vertex 70 FTIR spectrometer with a mercury cadmium telluride (MCT) detector cooled with liquid nitrogen. The adsorption behavior of CO_2 on the 15CuCe catalyst was investigated by *in situ* DRIFTS measurement at 300 °C. Prior to the *in situ* DRIFTS test, 30 mg sample was pretreated at 600 °C for 60 min under 5% H_2/Ar mixed gas. The background spectra were collected under N_2 atmosphere at 4 cm^{-1} resolution at 300 °C. The mixed gas consisted of 2% CO_2/Ar and was introduced into the chamber after the collection of background spectrum. Continuous recording of the IR profiles was maintained for 5 min. As for the RWGS conditions, after background acquisition, the reaction gas with 15% CO_2/30% H_2/55% N_2 is introduced into the *in situ* chamber. All DRIFTS results were analyzed by using OPUS software.

The density functional theory (DFT) calculations: The heterogeneous catalysis simulations were performed using the Vienna Ab initio Simulation Package[3–7]. The spin-unrestricted geometry optimizations were calculated under Perdew−Burke−Ernzerhof (PBE) exchange−correlation functional

and the projector augmented-wave (PAW) pseudopotential[7–9]. The cutoff energy for the plane wave basis was set to 400 eV for all atoms. The Hubbard U term (DFT+U) was applied to Ce 4f orbitals, and 4.5 eV was chosen for the value of U, in accordance with previous research[10–11]. The constructed CeO_2 {111} model was composed of a 12-layer slab (Ce 4 layers and O 8 layers; total 48 Ce and 96 O atoms) as well as a vacuum layer of 15 Å. To simulate the Cu/CeO_2 composite catalyst, ten Cu atoms (2 layers) were loaded to the CeO_2 {111} model. The convergence criterion of the electronic self-consistency (SC) loop was less than 1.0×10^{-6} eV, and the completion criterion of the ionic relaxation loop was that the norms of all the forces were below 0.02 eV. The constructed CeO_2 {111} model was composed of a 12-layer slab (Ce four layers and O eight layers), meanwhile, the total numbers of Ce and O atoms were 48 and 96 in turn. To accurately simulate the properties of bulk phase, the coordinates of the bottom three layers (Ce one layer and O two layers) were fixed during structure optimization processes. The top nine layers were fully relaxed, and the thickness of the vacuum layer was set to 15 Å. Ten Cu atoms were loaded to the CeO_2 {111} model in order to simulate the Cu/CeO_2 composite catalyst. The initial structure of the 2-layer Cu cluster was derived from Cu {111} surface, i.e., seven Cu atoms were situated on the under layer and the other three Cu atoms were situated on the upper layer. The bonds form between CeO_2 {111} and the Cu cluster was Ce–O–Cu. The lattice parameters of the modeling Cu/CeO_2 surface were a = 15.4131 Å, b = 11.5598 Å, c = 26.0116 Å, α = 90°, β = 90° and γ = 120°.

Supplementary Figures:

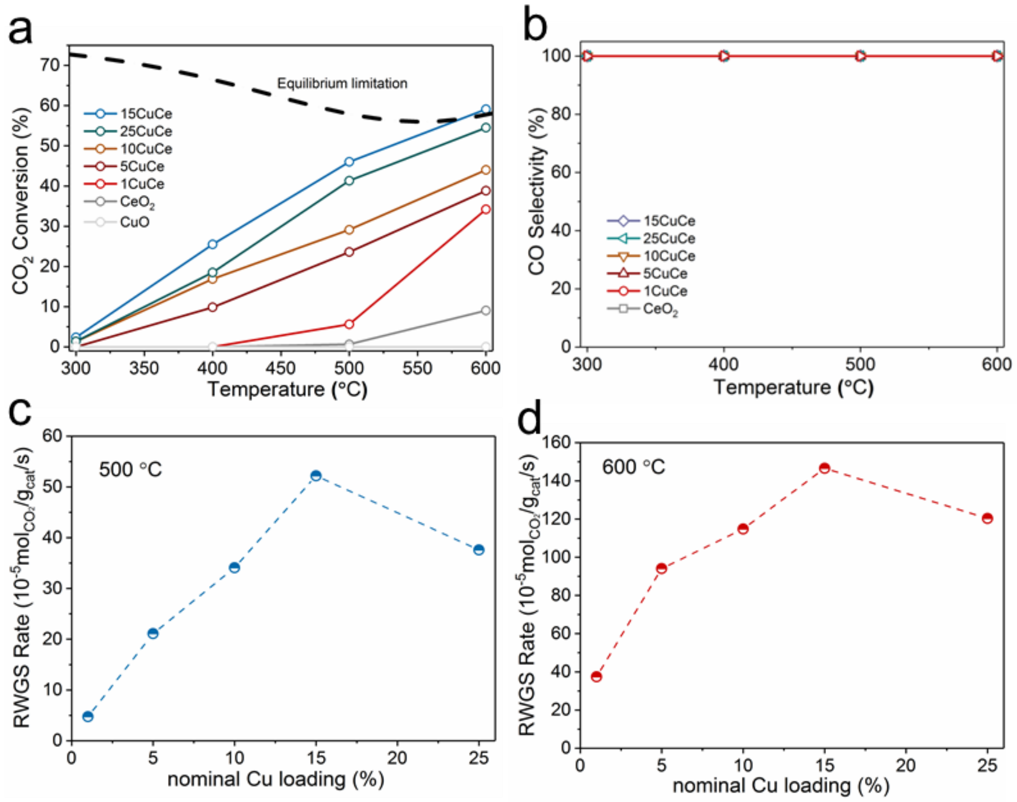

Supplementary Figure 1 | (a) CO_2 conversion over different catalysts and equilibrium limitation. (b) CO selectivity of CeO_2 support and all Cu/CeO_2 samples in RWGS reaction. (c, d) CO_2 reaction rates as a function of Cu content at 500 ºC and 600 ºC.

As shown in Supplementary Figure 1a, throughout the testing process, pure CuO has no activity, indicating that CuO particles (tens to hundreds of nanometers) could not catalyze this reaction.

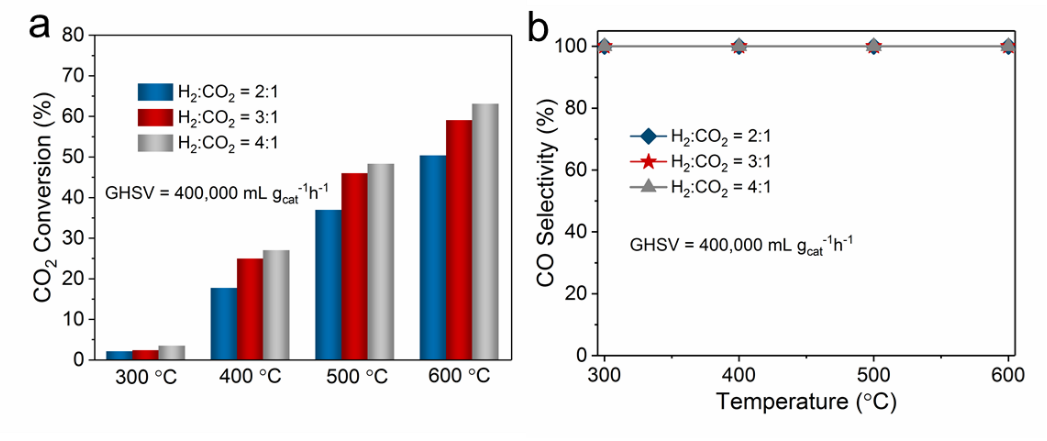

Supplementary Figure 2 | Catalytic performances for the 15CuCe catalyst at H_2:CO_2 ratio of 2:1, 3:1, and 4:1.(a) CO_2 conversion and (b) CO selectivity.

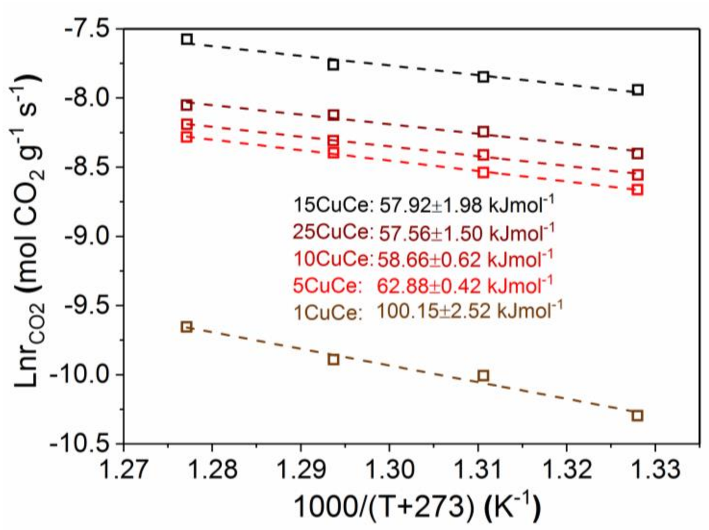

Supplementary Figure 3 | Arrhenius plots of RWGS reaction on Cu/CeO_2 catalysts.

Supplementary Figure 3 exhibited that the increase of copper content reduces the apparent activation energy, suggesting CO_2 reduction reaction is promoted with the aid of copper.

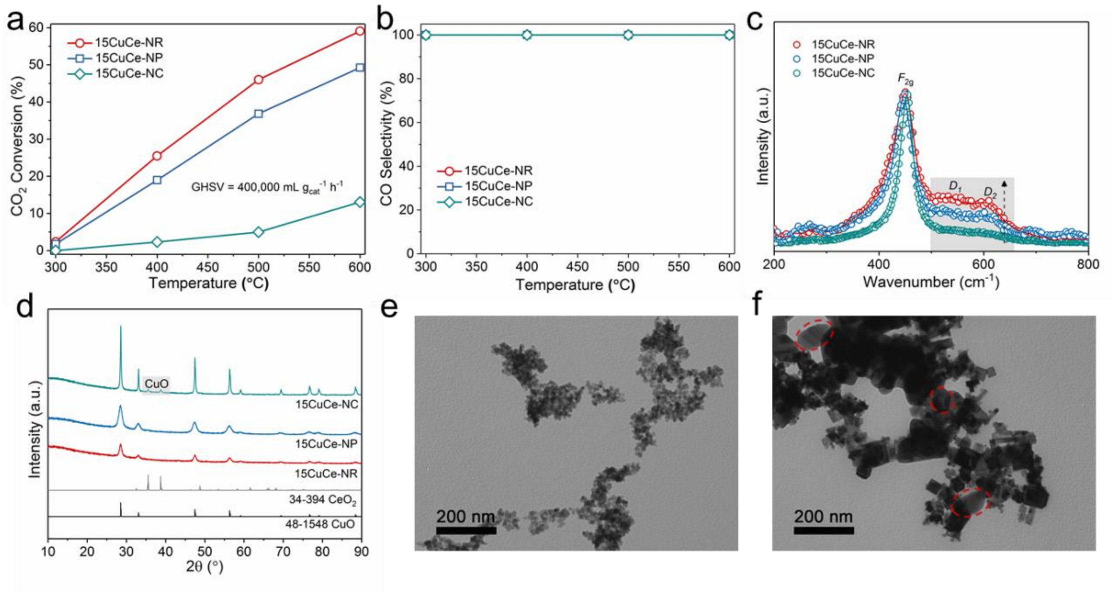

Supplementary Figure 4 | (a) CO_2 conversion and (b) CO selectivity of the 15CuCe-NR、15CuCe-NP and 15CuCe-NC catalysts. (c,d) Raman spectra and XRD patterns over the 15CuCe-NR、15CuCe-NP and 15CuCe-NC catalysts, respectively. (e,f) TEM pictures of the fresh 15CuCe-NP and 15CuCe-NC catalysts, respectively.

As illustrated in Supplementary Figure 4a, the CO_2 conversion rate ranked in the order of 15CuCe-NR > 15CuCe-NP > 15CuCe-NC. And all three catalysts showed 100% CO selectivity. From the Raman results of the fresh catalysts (Supplementary Figure 4c), the 15CuCe-NR catalyst had stronger oxygen vacancy peaks than the 15CuCe-NC and 15CuCe-NP catalysts. Higher oxygen vacancy concentration is favorable to the RWGS reaction. And as shown in the XRD results of the fresh copper–ceria catalysts with different morphologic ceria supports (Supplementary Figure 4d), the 15CuCe-NC catalyst shown more distinct diffraction peaks of CuO, which indicated the copper species had worse dispersion on ceria nanocube, causing the much inferior catalytic performance. The TEM pictures also reflected that there were many copper agglomerations (labeled by circle in red) on the ceria nanocube, but not on ceria nanoparticle. Highly dispersed copper species and higher concentration of oxygen vacancies made the 15CuCe-NR had the best initial RWGS reaction performance.

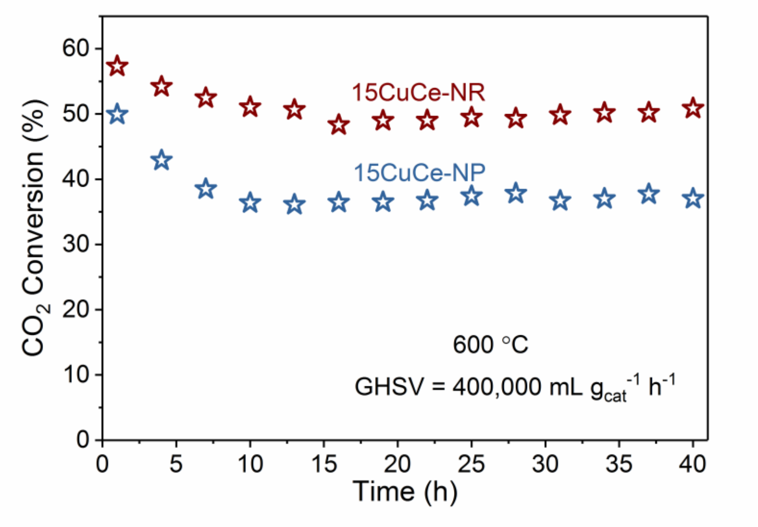

Supplementary Figure 5 | The stability tests of the 15CuCe-NP and 15CuCe-NR catalysts.

The 15CuCe-NP catalyst lost about 30% of its initial activity within the 40 h stability test. As shown in Supplementary Table 1, the 15CuCe-NP catalyst suffered more severe sintering, resulting in a significant decrease of specific surface area than that of the 15CuCe-NR catalyst, which caused an inferior activity.

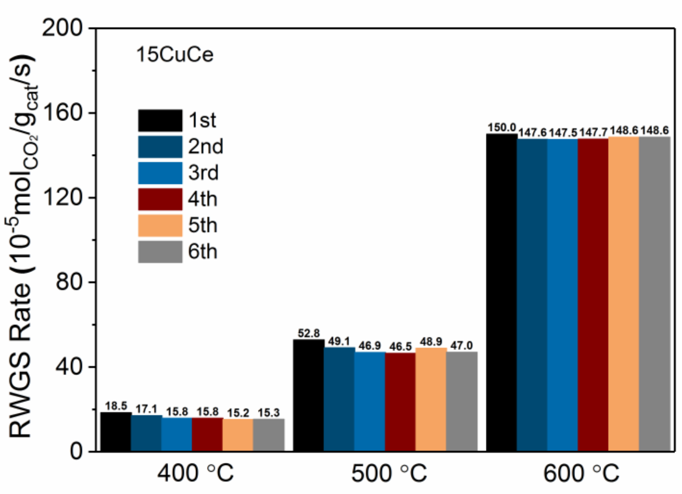

Supplementary Figure 6 | Reaction rates over the 15CuCe catalyst for six start-up cool down cycles.

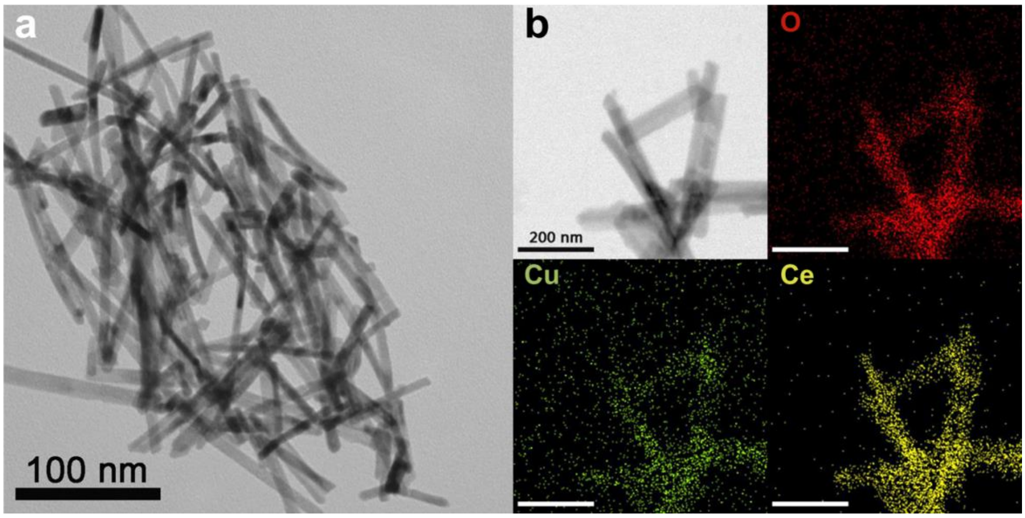

Supplementary Figure 7 | (a) TEM and (b) STEM images with element mapping results of the fresh 15CuCe catalyst.

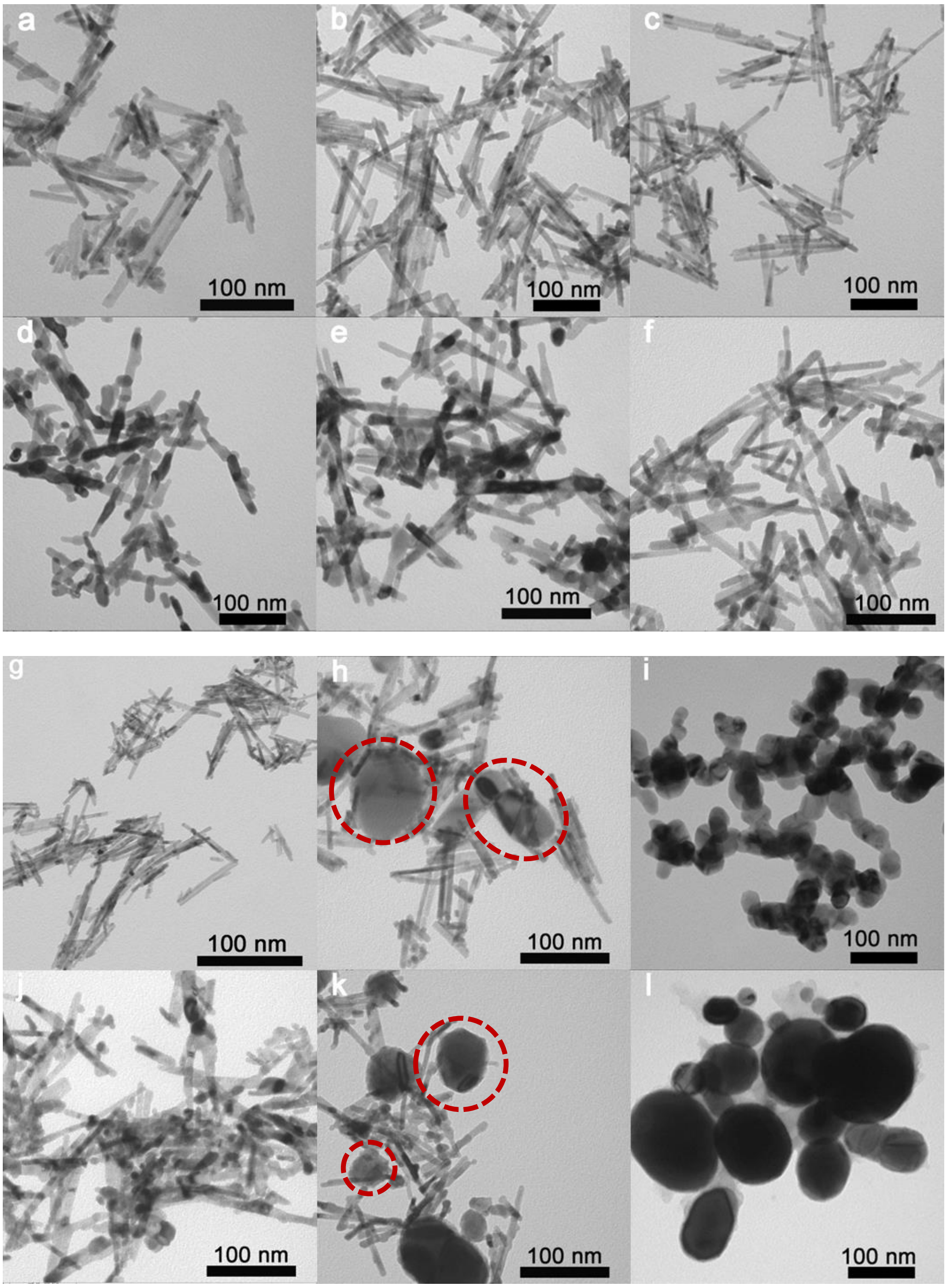

**Supplementary Figure 8 | ** TEM images of Cu/CeO_2 catalysts. TEM images of the fresh and used catalysts after various temperature test. (a, d) 1CuCe; (b, e) 5CuCe; (c, f) 10CuCe; (g, j) 15CuCe; (h, k) 25CuCe; (i, l) pure CuO.

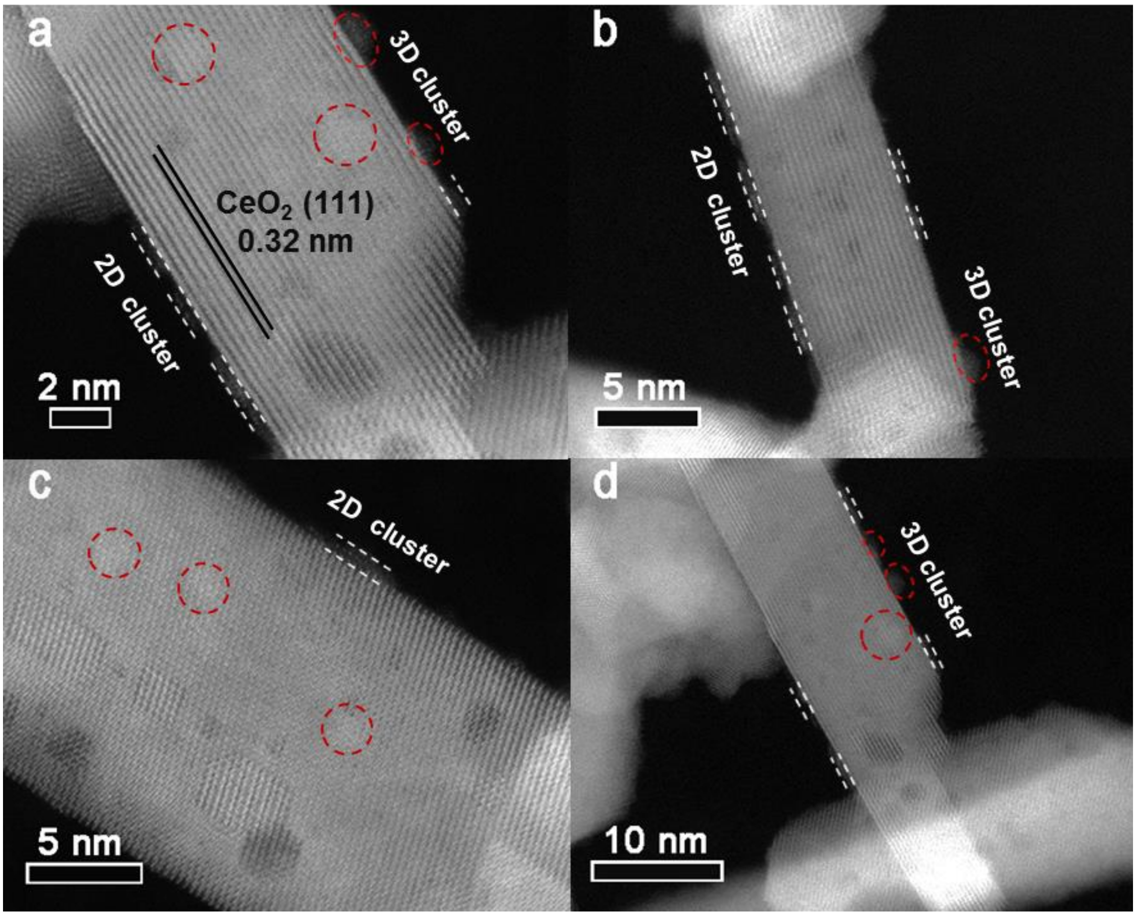

Supplementary Figure 9 | (a, b, c, d) HAADF-STEM images of the pre-reduced 15CuCe catalyst.

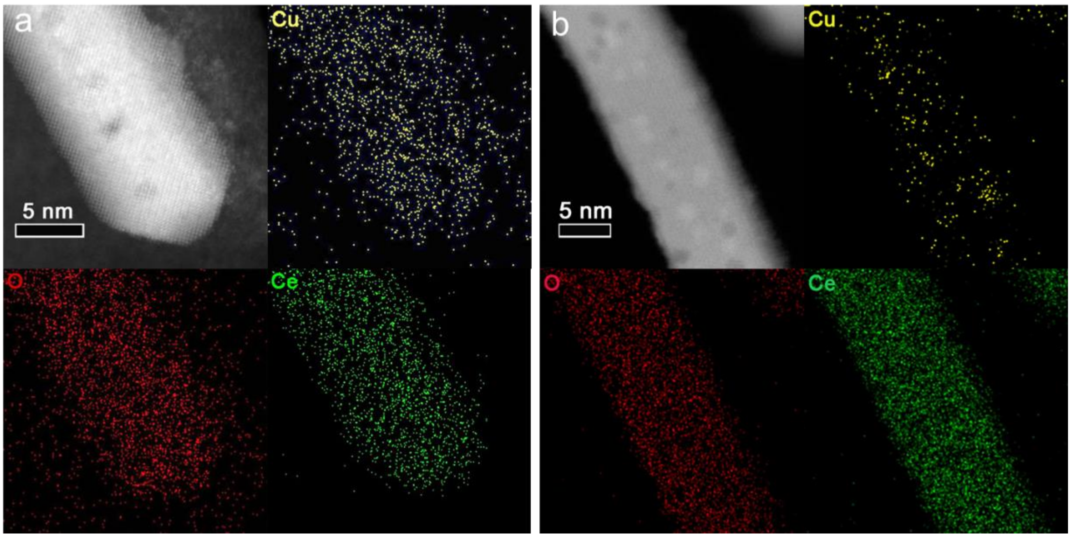

Supplementary Figure 10 | (a, b) STEM images with element mapping images about the partially sintered ceria support of the 15CuCe catalyst after 70 h stability test.

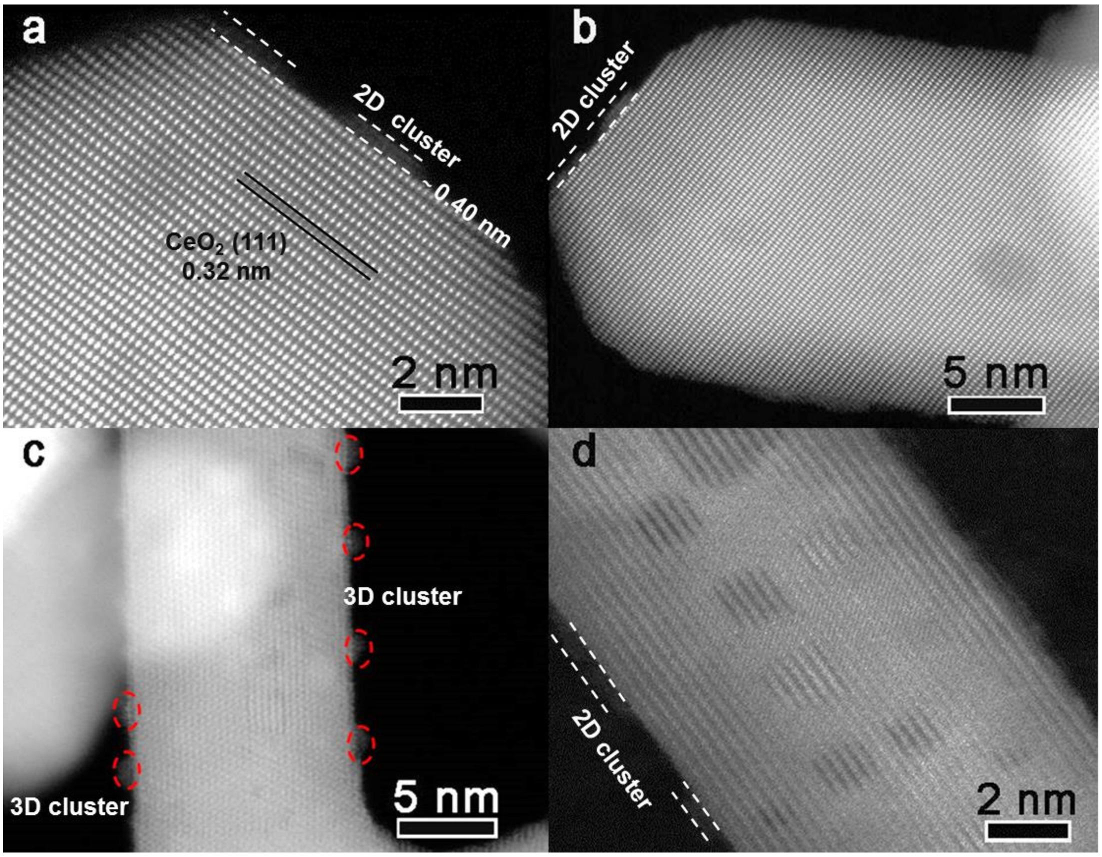

Supplementary Figure 11 | (a, b, c, d) HAADF-STEM images of the used 15CuCe catalyst after 70 h stability test.

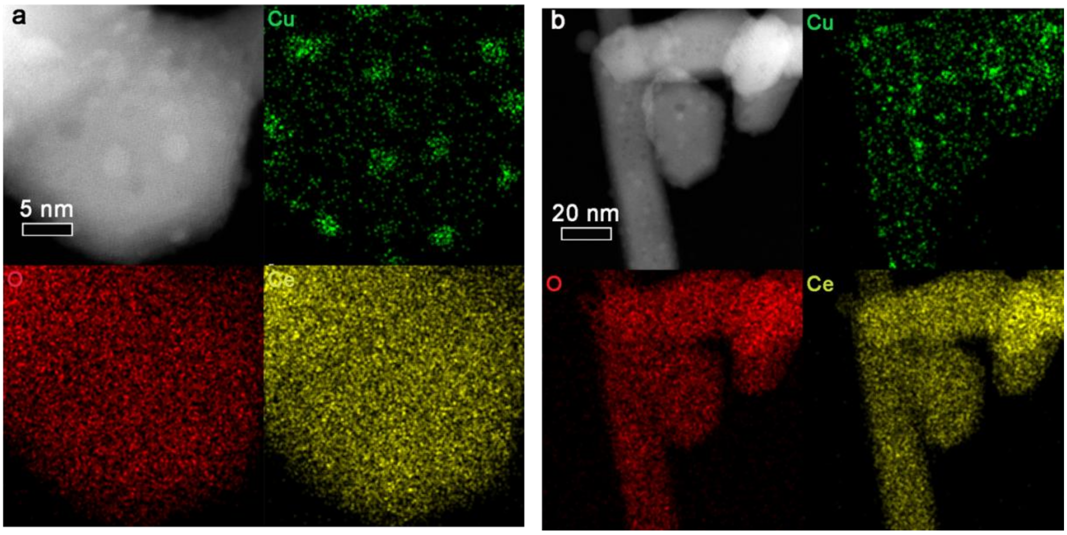

Supplementary Figure 12 | (a, b) STEM images with element mapping images of the 15CuCe catalyst after 240 h stability test.

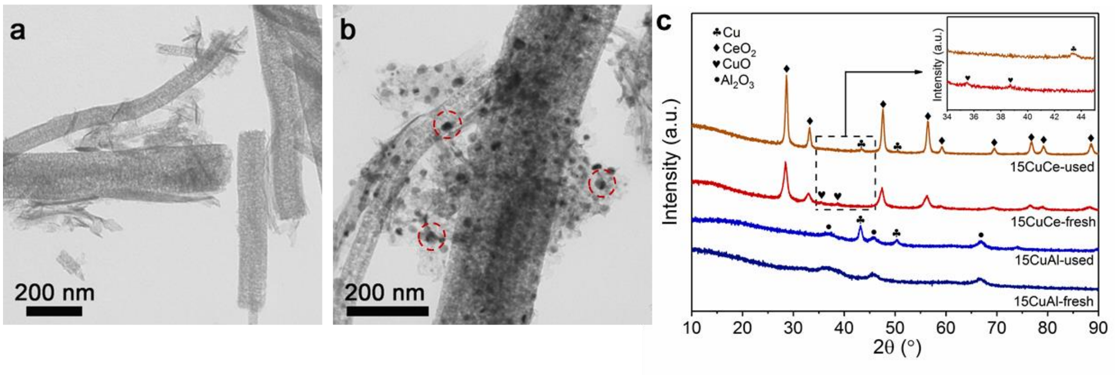

Supplementary Figure 13 | TEM images of (a) the fresh and (b) used Cu/Al_2O_3 catalyst. (c) XRD patterns of the fresh and used catalysts.

As shown in Supplementary Figure 12, no agglomerated copper species were found for the fresh 15CuAl catalyst. And there were also no diffraction peaks of copper specise in the XRD result of the fresh 15CuAl catalyst. Above experimental results indicated that the copper species was highly dispersed for the fresh 15CuAl catalyst.

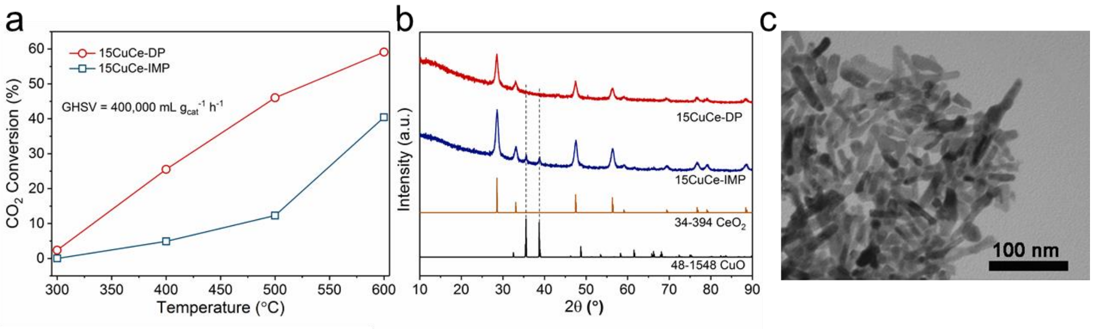

Supplementary Figure 14 | (a) CO_2 conversion of the 15CuCe catalysts prepared by DP and IMP methods. (b) XRD and (c) TEM results of the 15CuCe-IMP catalyst, respectively.

As shown in Supplementary Figure 13a, the 15CuCe-IMP exhibited much lower RWGS conversion than that the 15CuCe prepared by DP method. The distinct diffraction peaks of CuO in the XRD result (Supplementary Figure 13b) indicated the worse dispersion of copper species for the fresh 15CuCe-IMP sample. And the TEM picture showed that for the fresh 15CuCe-IMP sample (Supplementary Figure 13c), the ceria nanorod suffered more severe sintering during the preparation of catalyst, causing the decrease of the specific surface area (60.3 $m^2 \cdot g^{-1}$). The interaction between copper and ceria could not only stabilize the active metal, but also stabilize the ceria support. The copper species with poor dispersion also accelerated the sintering of ceria. Above data demonstrated for the copper–ceria catalyst, the dispersion degree of copper species had an important effect on the activity and structural stability. From the perspective of catalyst synthesis methods, copper was easier to be anchored on the ceria support by DP method rather than IMP method.

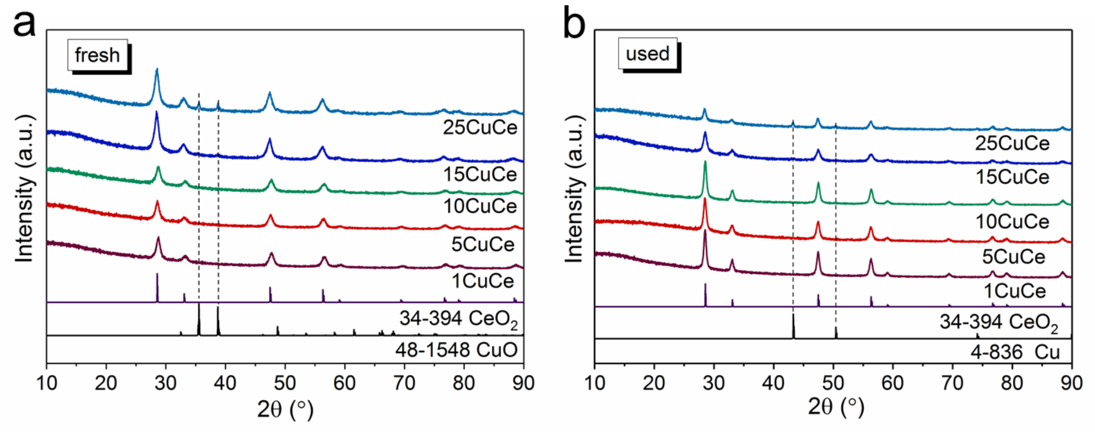

Supplementary Figure 15 | XRD patterns of (a) fresh and (b) used Cu/CeO_2 catalysts.

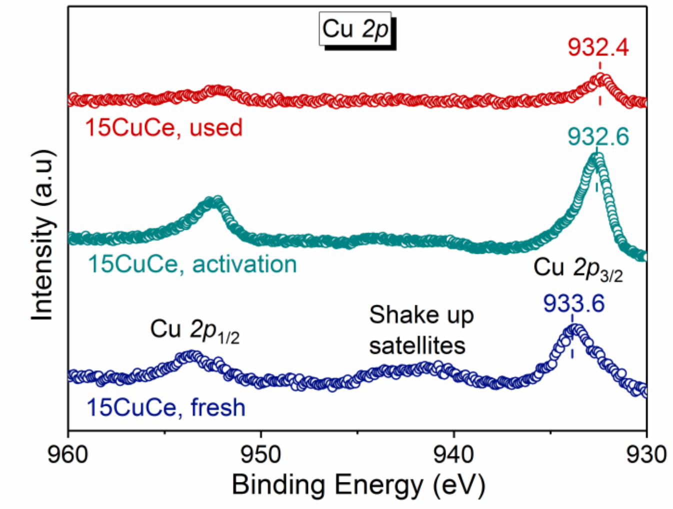

Supplementary Figure 16 | Cu *2p* XPS results of the fresh, pre-reduced and used 15CuCe catalysts.

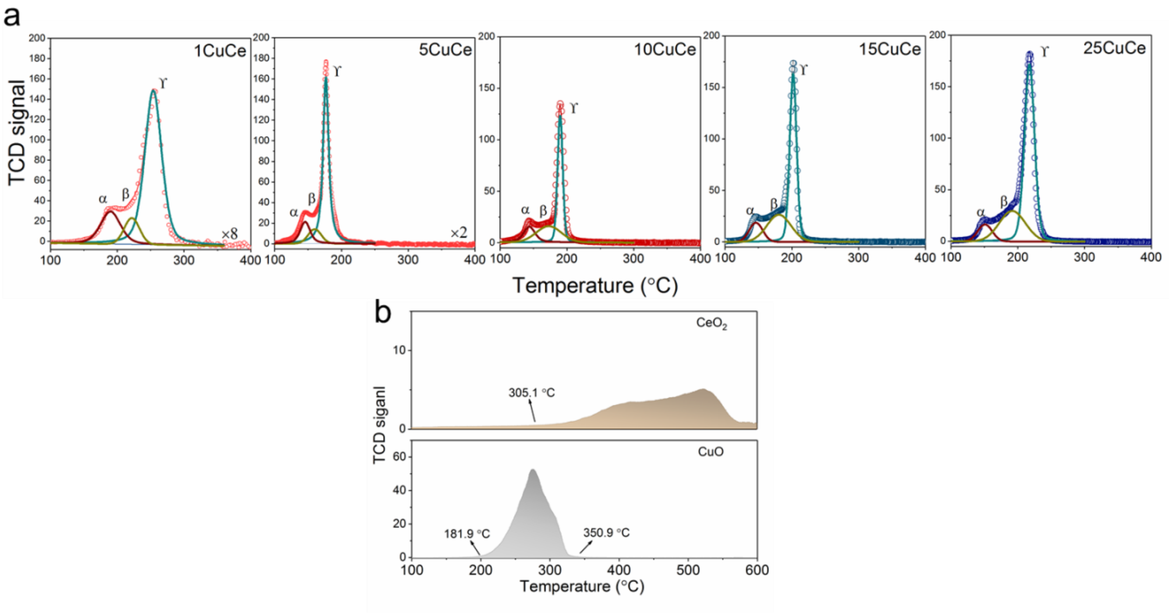

Supplementary Figure 17 | Profiles of the H_2-TPR for (a) all the copper-ceria catalysts and (b) CuO and CeO_2 samples.

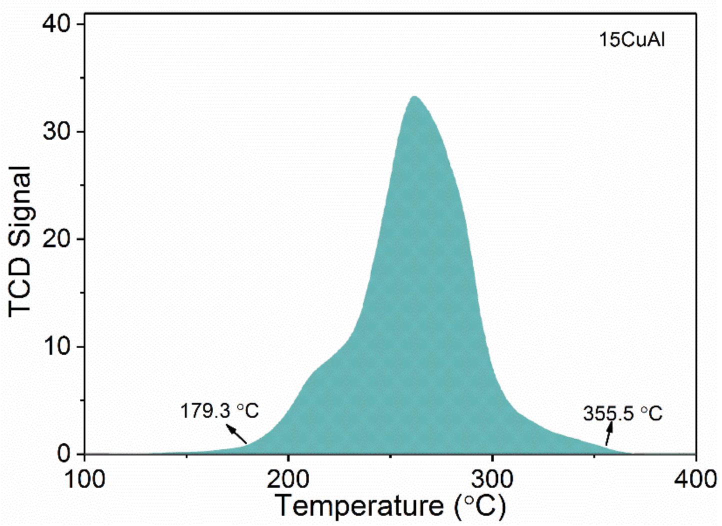

Supplementary Figure 18 | H_2-TPR over the 15Cu/Al_2O_3 catalyst.

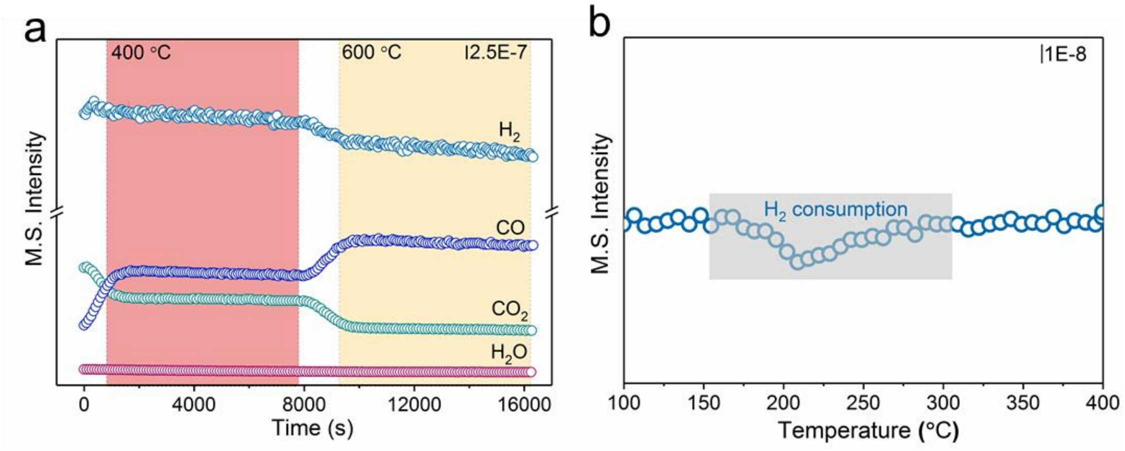

Supplementary Figure 19 | (**a**) TPSR result of the 15CuCe catalyst; (**b**) H_2-TPR result of 15CuCe catalyst following the TPSR test.

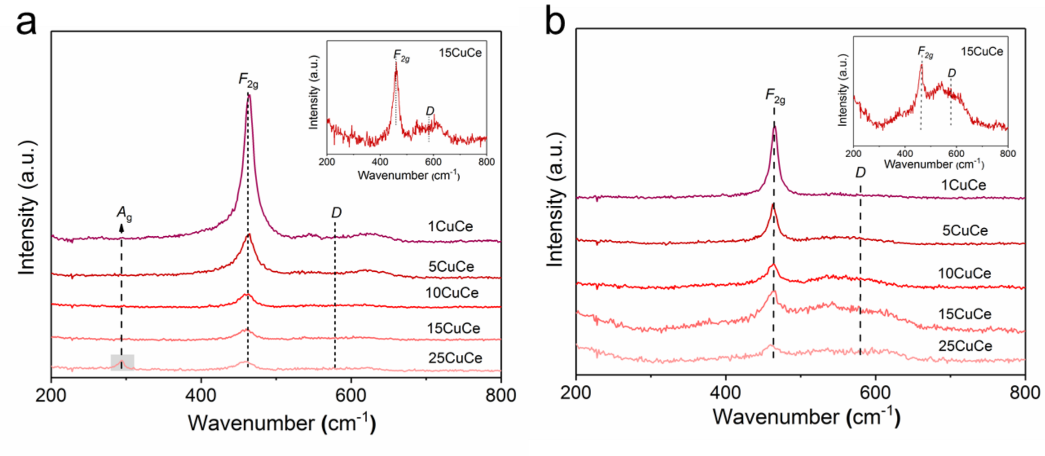

Supplementary Figure 20 | Raman spectra over (a) fresh and (b) used Cu/CeO_2 catalysts.

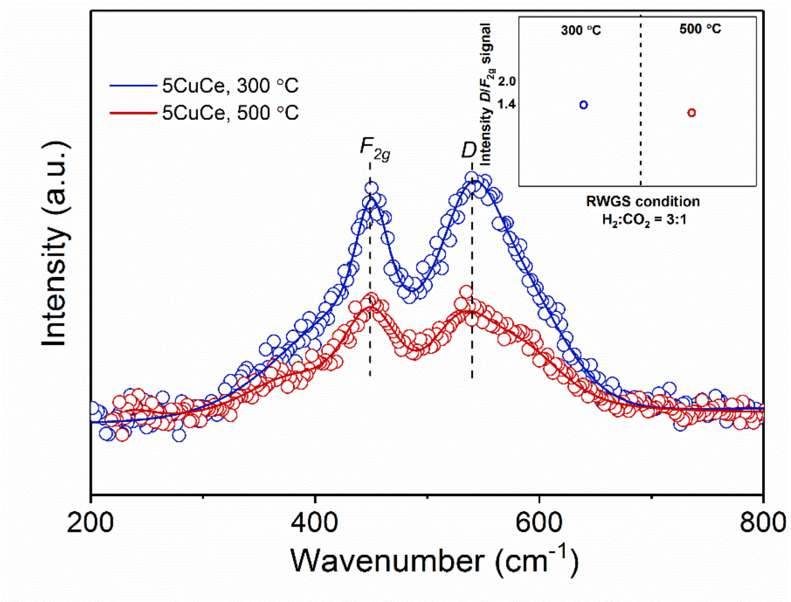

Supplementary Figure 21 | *In situ* Raman spectra under reaction process over the 5CuCe catalyst.

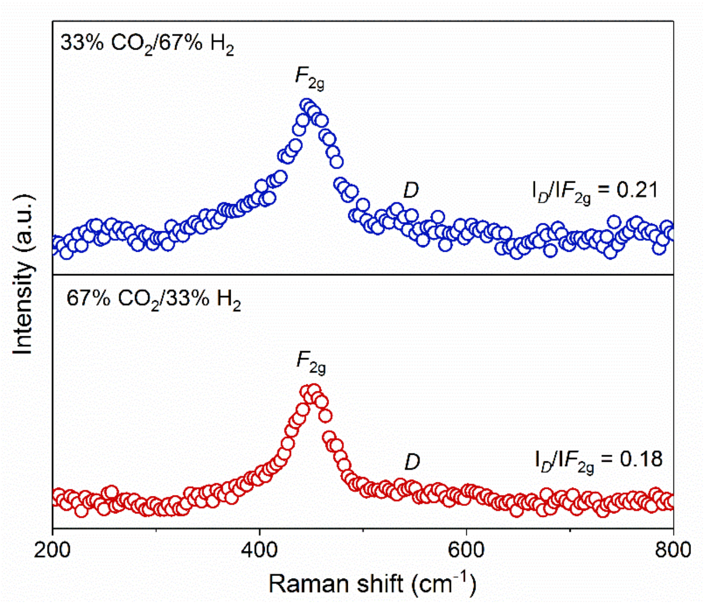

Supplementary Figure 22 | *In situ* Raman spectra over CeO_2 support under different atmosphere at 500 ºC.

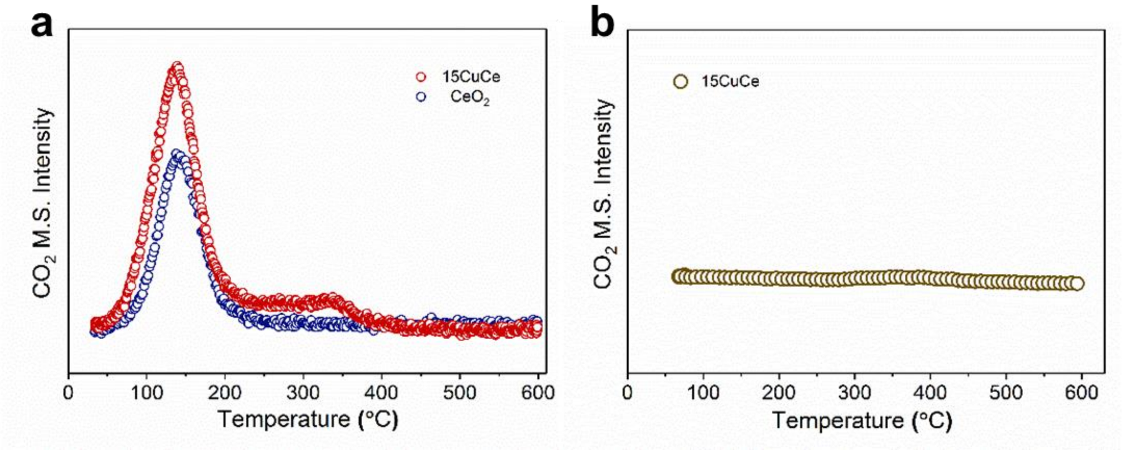

Supplementary Figure 23 | (a) CO_2-TPD profiles of CeO_2 and the 15CuCe catalyst. (b) Heating treatment with Ar gas after H_2 pretreatment without CO_2 pre-adsorption.

As shown in Supplementary Figure 23a, loading copper on ceria facilitates the adsorption of CO_2. And in Supplementary Figure 23b, no desorption signal of CO_2 was observed, indicating that the H_2 pretreatment process could remove the carbonate species formed on the catalyst surface in the air.

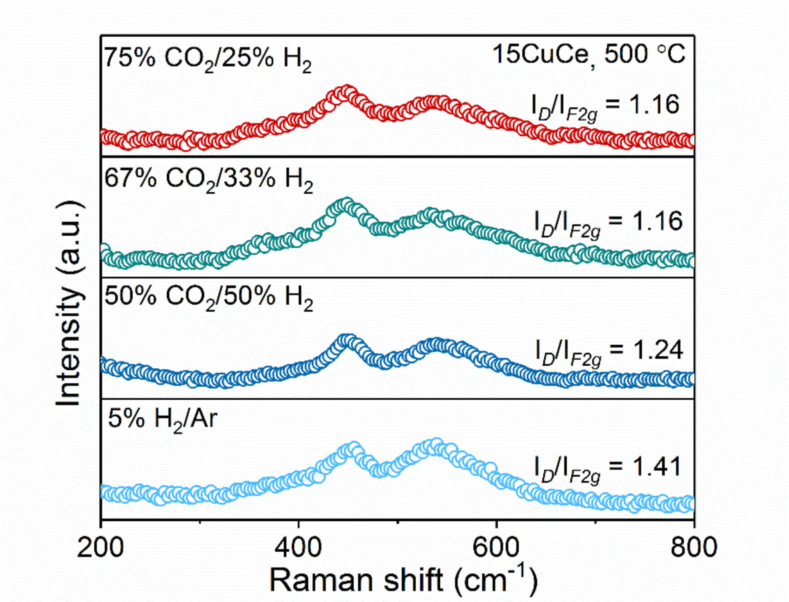

Supplementary Figure 24 | *In situ* Raman spectra of the 15CuCe catalyst under different atmosphere at 500 ºC.

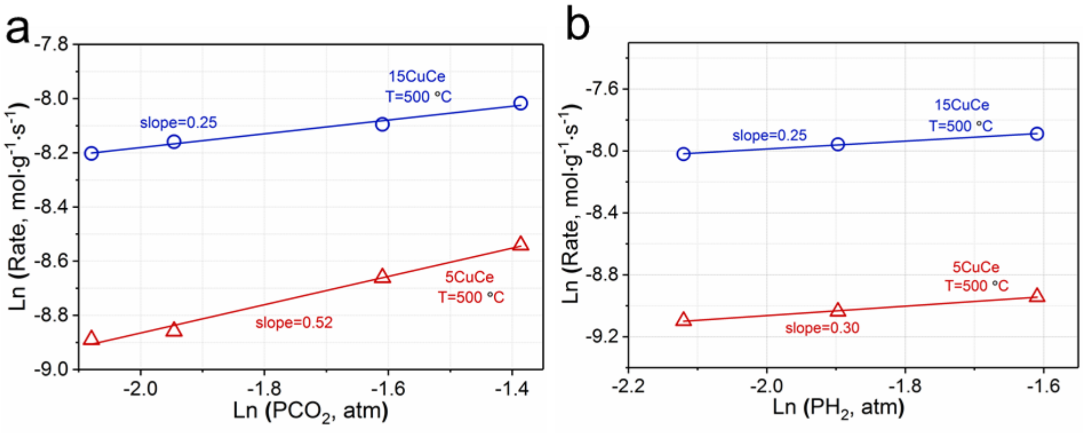

Supplementary Figure 25 | Kinetic order of (a) CO_2 and (b) H_2 on the 5CuCe and 15CuCe catalysts at 500 ºC.

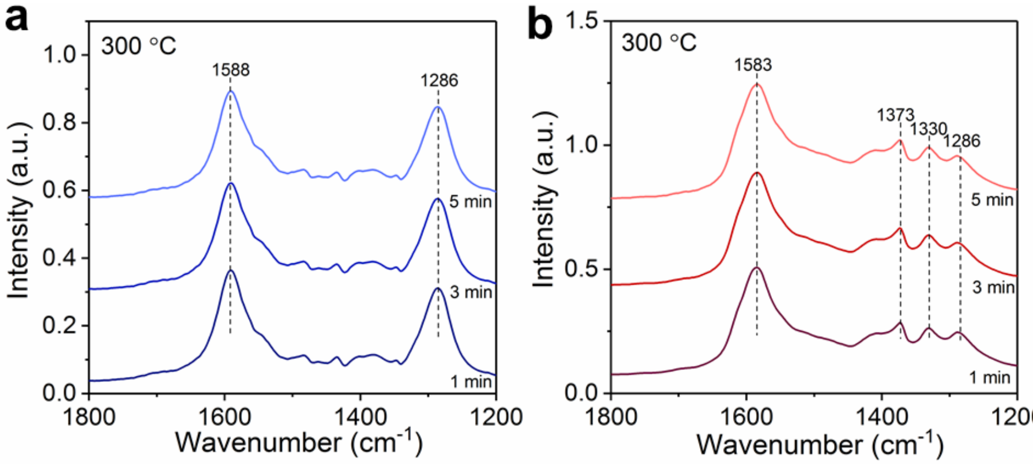

Supplementary Figure 26 | *In Situ* DRIFTS spectra of the 15CuCe catalyst during (a) CO_2 treatment and (b) RWGS reaction conditions at 300 ºC.

Supplementary Tables:

Supplementary Table 1. BET specific surface areas of various copper–ceria catalysts.

Catalyst	S_{BET} ($m^2 \cdot g_{cat}^{-1}$)
15CuCe-NR	$^{\alpha}$83.3,$^{\beta}$44.1
15CuCe-NP	$^{\alpha}$82.3, $^{\gamma}$27.9
15CuCe-NC	$^{\alpha}$23.3
15CuCe-IMP	$^{\alpha}$60.3

Note: (α) fresh catalysts. (β, γ) used catalysts after 70 h and 40 h RWGS reaction, respectively.

Supplementary Table 2. The single-point energies of the constructed surface models and intermediates.

Structures	E / eV	Structures	E / eV
V_O-A	-1218.133569	V_O-A + CO_2	-1243.052508
V_O-B	-1217.825478	V_O-B + CO_2	-1241.988960
V_O-C	-1217.889766	V_O-C + CO_2	-1242.117825
V_O-D	-1217.569036	V_O-D + CO_2	-1242.113886
V_O-E	-1218.779069	V_O-E + CO_2	-1243.594793
V_O/CeO_2 {111}	-1184.992895	V_O/CeO_2 {111} + CO_2	-1207.824954
IMA1	-1243.052508	IMA2	-1250.479925
IMA3	-1250.915264	IMA4	-1250.805777
IMA5	-1249.218921	IMA6	-1234.602513
IMA3-I	-1250.773474	IMA4-I	-1250.782227
IMA3-II	-1250.717540	IMA4-II	-1250.340765
CO_2	-23.014969	H_2	-6.762122
H_2O	-14.232397		

Supplementary References

1. Yu, W. et al. Construction of Active Site in a Sintered Copper-Ceria Nanorod Catalyst. *J. Am. Chem. Soc.* **141,** 17548–17557 (2019).
2. Yan, C. et al. Shape-Selective Synthesis and Oxygen Storage Behavior of Ceria Nanopolyhedra, nanorods and Nanocubes. *J. Phys. Chem. B* **109**, 24380–24385 (2005).
3. Kresse G., Hafner J. Ab initio molecular dynamics for liquid metals. *Phys. Rev. B* **47,** 558−561 (1993).
4. Kresse G., Furthmuller J. Efficient iterative schemes for ab initio total-energy calculations using a plane-wave basis set. *Phys. Rev. B* **54,** 11169−11186 (1996**)**.
5. Kresse G., Hafner J. Ab initio molecular-dynamics simulation of the liquid-metal-amorphous-semiconductor transition in germanium. *Phys. Rev. B* **49,** 14251−14269 (1994**)**.
6. Kresse G., Joubert D. From ultrasoft pseudopotentials to the projector augmented-wave method. *Phys. Rev. B* **59,** 1758−1775 (1999).
7. Perdew J. P., Burke K., Ernzerhof M. Generalized gradient approximation made simple. *Phys. Rev. Lett* **77,** 3865−3868 (1996).
8. Blöchl P.E. Projector augmented-wave method. *Phys. Rev. B* **50,** 17953−17979 (1994).
9. Saito M., Roberts C. A., Ling C. DFT+U study of the adsorption and oxidation of an iron oxide cluster on CeO_2 support. *J. Phys. Chem. C* **119,** 17202−17208 (2015).
10. Liu Y. L., Li H. Y., Yu J., Mao D. S., Lu G. Z. Electronic storage capacity of ceria: role of peroxide in Aux supported on CeO_2 (111) facet and CO adsorption. *Phys. Chem. Chem. Phys.***17,** 27758−27768 (2015).
11. Zhang J., Gong X. Q., Lu G. Z. Catalytic activities of CeO_2 (110)–2×1 reconstructed surface. *Surf. Sci.* **632**, 164−173 (2015) .
